# Research progress on theaflavins: efficacy, formation, and preparation

**DOI:** 10.1080/16546628.2017.1344521

**Published:** 2017-07-03

**Authors:** Hua-Feng He

**Affiliations:** ^a^ Key Laboratory of Tea Processing Engineering of Zhejiang Province, Tea Research Institute, Chinese Academy of Agricultural Sciences, HangZhou, China; ^b^ National Engineering Technology Research Center for Tea Industry, HangZhou, China

**Keywords:** Theaflavins, bio-activity, formation, preparation

## Abstract

**Background**: Theaflavins (TFs) are a category of natural compounds characterized with the benzotropolone skeleton. The prominent benefits of TFs have been well documented. Amount of research were conducted and excellent achievements were disclosed during the past years. However, as far as we know, there is no comprehensive review about TFs.

**Scope and approach**: This review summarized the recent research progress. The activity of TFs on anti-oxidation, anti-mutagenicity, hypolipidemic, anti-inflammatory, anti-cancer, anti-viral effect as well as the epidemiological cure were sorted. Converging pioneer literature and deduction, the underlying formation mechanism of TFs was proposed. Subsequently, acquisition of TFs was pointed out to be the fundament for further research. Accelerated by enzyme, bio-synthesis of TFs were reviewed simultaneously. At the end, employing modern analysis instrument and technology, isolations of TFs were enumerated.

**Key findings and conclusions**: Structure of the skeleton as well as functional groups were paramount related with the bio-activity of TFs. Meanwhile, oxidation pathway of two catechin molecules to form TFs were hypothesized. Also, ascertainment of the several therapeutic efficiency of the family members of TFs would be the next step in the future.

## Introduction

**Figure 1.**
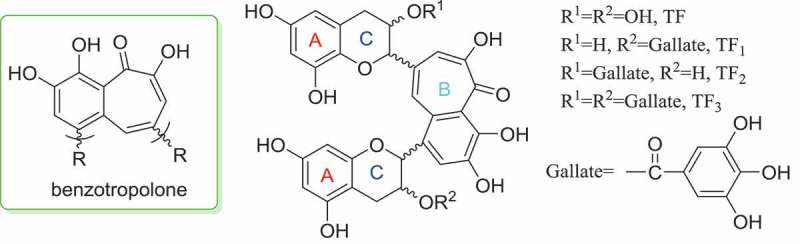
Chemical structure of theaflavins.

Originally found by Professor E.A.H. Roberts et al. [1], theaflavins (TFs) [2], characterized with the structure of benzotropolone as shown in Figure 1, were presented extensively in fermentative tea [3]. Due to their therapeutic attributions, TFs were regarded as the ‘golden molecules’ separated from tea (*Camellia sinensis*, fam. Theaceae). Meanwhile, TFs were also the crucial molecules that decide the quality and grade of black tea, the most consumed tea all over the world [4]. As the smallest tea-pigments, TFs were clarified to have positive correlation with the liquid color of black tea [5,6]. On the other hand, TFs also could accelerate the mellow and fresh degree of black tea infusions. Sensory analysis revealed that TFs imparted a mouth-coating, astringent, and long-lasting oral sensation at the back of the throat. It was demonstrated that TFs had by far lower oral thresholds than the astringent catechins, and accounted for less than 0.1% of the overall astringency of the teas investigated [7].

## Analysis of the structure-activity relationship (SAR) of TFs

When considering the activities of tea, anti-oxidation is the top of the list. As known, the anti-oxidative activity refers to polyphenols, such as epigallocatechin-3-gallate (EGCG). As the dimer of catechins, it is appropriate that TFs possess excellent anti-oxidative activity [8]. Generally, the existence of double bond at C_2_-C_3_ as well as the 4-OH in the skeleton of flavonoids, which was conjugated with the benzene ring, would benefit the formation of a more stabilized intermediate, which may enhance the antioxidant ability of flavonoids to some degree [9]. Though, with steric hindrance, oxygen atoms on the chromane skeleton still showed electron-withdrawn effect strongly. Meanwhile, benzotropolone as well as the hydroxyl and carbonyl on it, could generate a more stable conjugated system than flavonoids. Based on these advantages, the hydroxyl on benzotropolone would be more active in the removal of radicals.

In the study, aimed to gain insights of the anti-oxidation of TFs, Nomura and co-workers [9], disclosed that the chromane ring as well as the hydroxyl on it played the initial role in the anti-oxidant system, rather than the benzotropolone moiety. The observation of two A-ring fission products, which were identified using high-field 1D and 2D NMR spectral analysis, highlighted that the initial site in the hydrogen peroxide oxidant system was the A-ring rather than the benzotropolone moiety [10].

In contrast, it is indicated that the gallate structures that existed in the module were positively correlated with the antioxidant ability of TFs, which is similar to the catechins [11]. Taking human LDL oxidation as a model, Leung et al. [12] demonstrated that TFs possessed at least the same antioxidant potency as catechins. The study of anti-oxidative activity of TFs conducted by Su et al. [13] showed that all of the four TFs were more powerful than BHT in the anti-oxidation; and the hierarchy is TF_3_ > TF_2_ = TF_1_ > TF. On the other hand, spectroscopic studies showed that all the TFs could chelate iron and copper ions, which would be helpful for the anti-oxidative mechanism research into TFs.

## Bio-activities of theaflavins

Among the molecules that separate from tea, TFs were the first one which were confirmed useful with the cure for hyperlipidemia as well as cardiovascular disease (CVD) [14–16]. Assays taken in vitro and in vivo indicated that TFs may suppress the inter- and intra-cellular expressions of gene proteins, inhibit the proliferation of cells and induce apoptosis effectively. In areas such as anti-microbial [17], hypolipidemic, anti-inflammatory, anti-mutagenicity [18], and anti-cancer, TFs showed excellent activity. Also, epidemiological studies suggested that TFs could be exploited to become a drug.

### Radical-scavenging activity

It is verified that conversion of catechins to TFs during fermentation when making black tea did not alter significantly their free radical-scavenging activity. Furthermore, TFs showed the ability to scavenge free radicals both in the aqueous and lipophilic phases. In protecting against H_2_O_2_-mediated damage in HPF-1 cells, which externalized as the hydroxyl radicals-scavenging ability, TFs showed more effective effects than EGCG. The order of 2, 2-diphenyl-1-picrylhydrazyl scavenging ability was TF_3_ > TF_2_ = TF_1_ > EGCG > TF [19,20]. Similarly, TF showed significant protection against the t-BHP induced oxidative stress, as evidenced by the decrease in protein carbonyl (PCO) and sulfhydryl group (-SH) content [21]. A randomized, double-blind, crossover study was conducted by Arent and co-workers [22], to exploit the effect of TFs on the responses of human subjects to acute anaerobic interval training. The result showed that consumption of TF-enriched black tea extract led to improved recovery and reduction in oxidative stress, and delayed onset muscle soreness responses to acute anaerobic intervals.

### Anti-mutagenicity activity

Studies on the response to benzo[*a*]pyrene (B[*a*]P), a known carcinogen, indicated that TFs had significant anti-clastogenic effects [23]. Compared with cultures that treated with B[*a*]P or aflatoxin B1 only, both chromosomal aberrations and micronuclei formation were observed with a significant decrease in the human lymphocyte cultures that were treated with either TF or TR. A similar result on B[*a*]P induced lung carcinogenesis was obtained [24]. Also, in salmonella assay taken in bone marrow cells of mice in vitro and in vivo, TFs showed the inhibition of mutagenicity and genotoxicity [25]. As well as enhanced serum testosterone levels, improved sperm characteristics, and abrogation of DNA damage, administration of TFs led to alleviation of Cd-induced damage in testis [26].

### Hypolipidemic activity

In respect to the lipase and amylase inhibitory activities, TFs were ascertained to be attributable [27]. Through blockage of NF-κB and JNK activation in intestinal epithelial cells, TF could suppress the expressions of lipopolysaccharide-induced intercellular adhesion molecule and vascular cell adhesion molecule [28]. On the other hand, by down-regulating epidermal growth factor and receptor/PI3K/Akt/Sp-1 signal transduction pathway, TFs suppressed the expression of fatty acid synthase, a key enzyme in lipogenesis. Both of these results bestowed TFs' hypolipidemic effects [29].

In the prevention of obesity, TFs significantly reduced lipid accumulation, suppressed fatty acid synthesis, and stimulated fatty acid oxidation, attenuated hepatic lipid accumulation through stimulating AMP-activated protein kinase [30]. Increased energy expenditure and expression of metabolic genes could be evidenced after oral administration of TFs [31,32]. All these suggested TFs potential application in functional foods and nutraceuticals for obesity management. Decreasing liver steatosis, oxidative stress, inflammation, and hepatocyte apoptosis by theaflavin treatment, Luo and co-workers [33], concluded that TFs also had protective effects against ischemia-reperfusion (I/R) injury in fatty livers.

### Anti-inflammatory activity

Expression of cytokine IL-6 may result in serious tissue injury and apoptosis. Decreasing the expression level of cytokine IL-6 during viral infection, TF derivatives exerted anti-inflammatory property [34]. Using a cell model for inflammatory response, Gosslau and co-workers [35], showed that TF_2_ suppressed the 12-*O*-tetradecanoylphorbol-13-acetate-induced COX-2 gene expression, and also down-regulated TNF-*α*, inducible nitric oxide synthase (iNOS), ICAM-1, and nuclear factor κB (NF-κB). As a similar mechanism, TF_3_ aided the protection of colonic inflammation [36]. Meanwhile, TFs significantly protected neurons from cerebral I/R injury by limiting leukocyte infiltration and expression of ICAM-1, and suppressing up-regulation of inflammatory-related pro-oxidative enzymes (iNOS and COX-2) in ischemic brain via reducing the phosphorylation of STAT-1 [37]. Inhibiting the activation of the epidermal growth factor receptor as well as decreasing the level of mucin 5AC, TFs relieved airway mucous hypersecretion, which might be valuable in the treatment of chronic airway inflammation [38].

Hosokawa and co-workers [39], investigated the effects of TF_3_ on CXC chemokine ligand 10 production from human gingival fibroblasts, and the results showed that TF_3_ had a dose dependent manner on the prevention of OSM-mediated CXCL10 production. Anandhan et al. [40] pointed out that therapeutic attenuation of neuro inflammation in Parkinson’s disease (PD) by TFs may provide a precious therapeutic strategy for the treatment of progressive neurodegenerative disease in the future. By the way, the ability to induce the secretion of the antimicrobial peptides hBDs by oral epithelial cells suggested TFs had a beneficial effect against periodontal disease [41].

### Anti-cancer activity

With respect to the effect on tumor cells, TFs, especially TF_3_, exhibited inhibitory activity on the extracellular signal transmission and cell proliferation [42]. By inducing cell shrinkage, membrane blebbing, and mitochondrial clustering, TFs triggered apoptosis of cancer cells such as mammary epithelial carcinoma cells [43] and leukemia cells [44].

Inhibition of NF-κB via p53–ROS crosstalk, as mentioned before, bestowed TFs' anti-migratory effect on cancer cells [45]. Except (−)-epicatechin, all of the tea polyphenols showed significant inhibition of cell growth and activator protein activity [46]. Furthermore, TF_3_ could inhibit the phosphorylation of extracellular signal-regulated kinase, such as the epidermal growth factor and PDGF receptors in A431 cells and mouse NIH3T3 fibroblast cells [47]. In the area of LNCap, TF_3_ showed inhibitory activity via suppressing the expression of the androgen receptor and lowering androgen-induced prostate-specific antigen secretion and fatty acid synthase protein levels [48,49]. In other words, TF_3_ was deemed to be a potential chemoprevention agents for prostate cancer. Among a set of pro-apoptotic genes, TF_2_ quickly induced the up-regulation of p53 and BAX, suggesting mitochondria as the primary target [35]. Gao et al. [50] showed that ascorbic acid enhanced the apoptosis of human lung adenocarcinoma SPC-A-1 cells and esophageal carcinoma Eca-109 cells induced by EGCG and TF_3_ via mitogen-activated protein kinases pathways. Due to the generation of H_2_O_2_, TFs revealed cytotoxicity of cells from the human oral cavity, which could be decreased with the presence of Co^2+^ or catalase. Fortunately, compared to normal cells, both malignant carcinoma cells and immortalized cells were more sensitive [51,52]. By the way, hydrolysis of theaflavin gallates mediated by salivary esterases indicated the possible use of theaflavins in the prevention of oral cancer and dental caries [53]. Without adversely affecting normal human epidermal keratinocyte cells, inhibition of A431 and A375 cell proliferation induced by TFs were reported. And the molecular mechanism predicted that TFs arrested the cell cycle through Bax translocation and inducing apoptosis via mitochondrial death cascade [23].

### Anti-viral effect

Epidemiological studies suggested that TF_3_ was an effective inhibitor of 3CL^pro^, related to severe acute respiratory syndrome (SARS) [54]. Evaluated with a neuraminidase activity assay, a hemagglutination inhibition assay, a real-time quantitative PCR assay for gene expression of HA, and a cytopathic effect reduction assay, TF derivatives might have a direct effect on viral particle infectivity, which was consistent with the inhibitory effects against the influenza virus [34]. TFs also exhibited potential activity in the prevention of HIV sexual transmission, which inhibited the infection by targeting the entry step [55,56]. As a microbicide to prevent HIV sexual transmission, vaginal gel formulation based on theaflavin derivatives had been evaluated [57]. Involving inhibition of the fluctuations of cytokines and maintenance of antioxidant status, TF_2_ and TF_3_ contributed to the prevention of oxazolone-induced type IV allergy in male ICR mice via percutaneous as well as oral administration [58]. On the other hand, TFs demonstrated strong anti-bacterial activity against clinical isolates of S. maltophilia and A. baumannii [59]. Using Vero and A549 cells as models, TFs showed a direct effect on the virions, that raised the HSV resistant strains [60].

### Other potential activities

In the prevention of CVD, Lorenz et al. [61] pointed out that TFs predominantly counterbalanced the lack of catechins in black tea, which resulted in the equally potent stimuli of NO production and vasodilation as green tea. More effectively than EGCG in the suppression of actin ring formation, TF_3_ inhibited the formation and differentiation of osteoclasts via inhibition of Matrix metalloproteinases MMPs. The result demonstrated that TF_3_ may be suitable agents or lead compounds for the treatment of bone resorption diseases [62]. Simultaneously, TFs showed a protective effect on dimethylnitrosamine-induced liver fibrosis [63]. Park et al. [64] and co-workers demonstrated that TFs had the ability to close the TJ route in Caco-2 cells, which resulted in the enhancement of the intestinal barrier. Thus, the ingestion of TFs would be expected to be therapeutic to bowel disease in the future. According to the research by Fukuda et al. [65], TFs could be developed as natural antagonists of AhR.

## Formation of theaflavins

**Figure 2.**
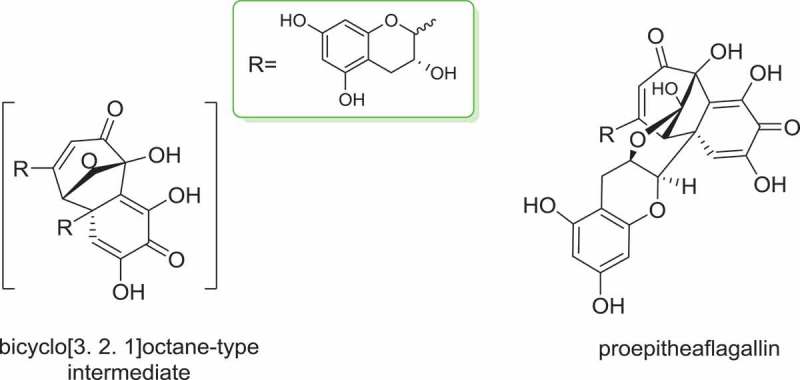
Intermediate isolated during the formation of theaflavin.

**Figure 3.**
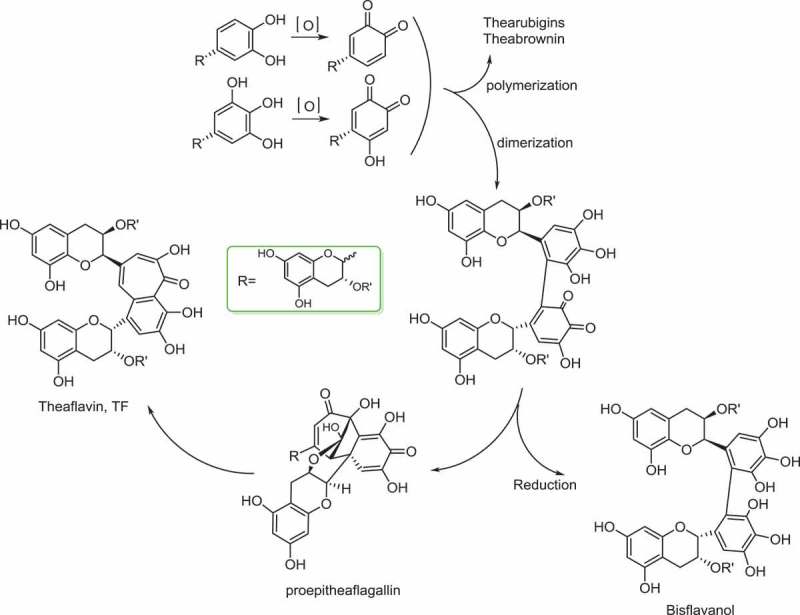
Underlying mechanism of the formation of theaflavins.

Sang et al. [66] disclosed that a benzotropolone skeleton is formed from co-oxidation of appropriate pairs of catechins, one with a vic-trihydroxy moiety, and the other with an ortho-dihydroxy structure. Isolation of the intermediate proepitheaflagallin, shown as in Figure 2, revealed that production of theaflavin was via a bicyclo[3.2.1]octane-type intermediate [67]. Based on this pioneering literature [68], and taking the endogenous and exogenous factors in consideration, the underlying formation mechanism of theaflavin related to enzymatic acceleration, and also physical-chemical transformation was hypothesized, as shown in Figure 3. With the mediation of enzymes, two catechin molecules were oxidized to quinone equally. Diphenol quinone was formed with the dimerization of the quinones. On the other hand, the polymerization of quinones would result in the formation of thearubigins as well as theabrowin. Sequentially, a bicyclo[3. 2. 1]octane-type intermediate, proepitheaflagallin was generated. It is noteworthy that proepitheaflagallin was an interactive quinone, the reduction of which would product bisflavanol. With further oxidization and dienone-phenol rearrangement, the motif of theaflavin was constructed.

Formation of theaflavin occurred via the reaction of a flavanol quinone with a non-quinone flavanol, in other words, only a dihydroxy-B-ring flavanol with a trihydroxy-B-ring flavanol gave a theaflavin upon enzymatic oxidation [69]. It is evidenced that unequal depletion rates of di- and trihydroxylated catechins led to a decline in total TFs and an increase in TB levels [5]. An equitable decline in both groups of catechins corresponded to a subsequent rise in theaflavins content. Decline in the catechins levels was much faster at higher temperatures. And this resulted in a shorter fermentation time to achieve maximum TF content.

**Figure 4.**
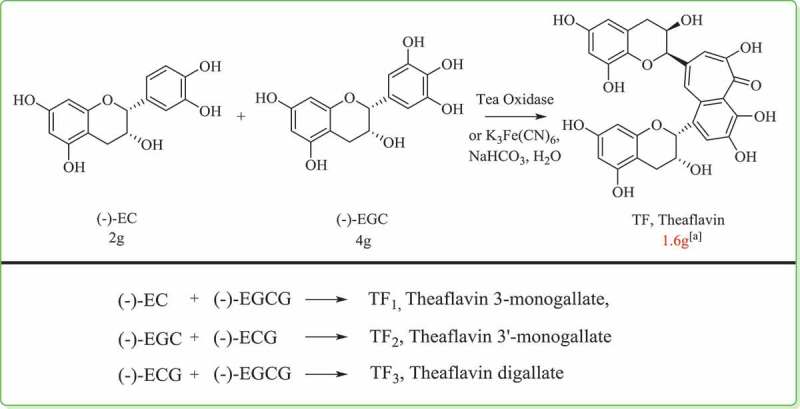
Generation of the four dominant theaflavins. ^[a]^ Reaction condition: 2 g of GC and 4 g of EC were dissolved in 600 ml of water, then 30 ml of an oxidizing reagent which was prepared by combining equal volume each of aqueous potassium ferricyanide (3.08 g in 10 ml) and aqueous sodium hydrogen carbonate (0.78 g in 10 ml) was added to the solution of catechins, dropwise under ice-cooling [70].

Resulting from the enzymatic oxidation of two catechin molecules, the family of TFs consisted of 25 members or more. TF, TF_1_, TF_2_ and TF_3_ dominated. Shown as Figure 4, we diagrammatized the production of these four dominating TFs. Taking EC, EGC, ECG and EGCG as precursors, TFs were generated by the dimerization of each two.

## Preparation of theaflavins

It is known that TFs account for 2–6% in the dry weight of solids in brewed black tea approximately. Biosynthesis would be an efficient route to obtain quantities of TFs. It is well known that polyphenol oxidase (PPO) and peroxidase (POD) are key enzymes in pigment generation during the process of making black tea. Model oxidation systems around PPO as well as POD were exploited to realize the biosynthesis of theaflavins. Using an immobilized polyphenol oxidase system, five critical variables, tea polyphenol concentrations, pH, aeration volumes, reaction time, and ratio of immobilized enzyme to the substrate, were optimized via response surface methodology at five levels and the highest theaflavin concentration obtained was 0.766 mg/ml [71]. With a conversion efficiency of 85%, about 14-fold increase over maximum achievable in normal black teas, TFs were bioprocessed on an immobilized tea PPO system in a cost effective manner [72].

Heterogeneous catalysis systems that utilize endogenous tea enzymes, PPO and POD, can successfully simulate the molecular changes that occurred during tea production [73]. Using the horseradish POD/H_2_O_2_ system, Sang et al. [66], reported the synthesis of 18 theaflavin derivatives. Pectinase enzymes isolated from Aspergillus spp., A. indicus, A. jluvus, and A. niveus were used for fermentation of tea leaves. Crude enzymes comprised of all enzymes, cellulase, hemicellulase (xylanase), proteinase, pectinase, etc., were more effective than the purified enzymes [74].

When it came to to obtaining TFs, Collier et al. [75] did some pioneering work. Tea samples were immersed with hot water, and the leach liquor was mixed with methanol and extracted with chloroform to remove the caffeine. Subsequently, the solvent was removed under vacuum, and the residue was extracted with ethyl acetate several times. With the removal of solvent, crude TFs could be obtained as orange-yellow solid.

Because of their low abundance and challenging purification procedure, previous research on TFs were focused on using mixtures. Alternatively, chemical synthesis could allow preparation of large quantities of pure compounds for biological assays. Furthermore, isomers of TFs as well as similar ramifications possessing the basic motif of benzotropolone skeleton distinguished with each other in the bio-activity. Intricate investigation was required to ascertain the therapeutic efficiency of the monomers within the theaflavin family. In respect to thermal and pH-dependent stability, it is concluded that GTC in green tea, in general, is more stable than TF in black tea. The lower the pH value of the sodium phosphate buffer, the greater the stability. When incubated in a buffer of the same pH, TF was much more unstable than GTC. Among the four TF derivatives, TF_3_ and TF_2_ had relatively slower rates of destruction than the other two [76]. Using reversed-phase high-performance liquid chromatography and capillary electrophoresis, simultaneous determination of catechins and theaflavin were achieved [77]. Together with preparative HPLC, separation of TFs could be achieved through high-speed countercurrent chromatography by gradient elution [78,79]. Cyclic voltammetry was also employed to characterize the phenolic compounds in green tea, oolong, black tea, and coffee [80].
